# Unusual Foreign Body Ingestion: A Fish Bone or a Bottle Cap?

**DOI:** 10.7759/cureus.92969

**Published:** 2025-09-22

**Authors:** Sandhu Gurveen, Jennifer Chew, Gloria S Kim, Lillian Chen

**Affiliations:** 1 Internal Medicine, University of California Los Angeles, Los Angeles, USA

**Keywords:** diagnostic work-up of ingestions, foreign body imaging, foreign body ingestion, ingestions, triage of ingestions

## Abstract

Foreign body ingestion is uncommon in adults but can pose significant risks. We present the case of a 55-year-old male patient who developed acute odynophagia after heavy alcohol intake. Radiographs revealed a metallic bottle cap lodged in the proximal esophagus. The patient was emergently referred for endoscopy, but initial endoscopic retrieval attempts were unsuccessful, requiring combined gastroenterology and otolaryngology intervention under general anesthesia. The object was removed, with only superficial ulcerations noted. The patient recovered with supportive care and was discharged without complications. This case highlights the importance of a detailed history, early imaging, and multidisciplinary management in high-risk foreign body ingestion.

## Introduction

While foreign body ingestions are far more common in the pediatric population, adult cases are rising. According to survey data from the National Electronic Injury Surveillance System in the United States, the prevalence of adult foreign body ingestion has increased from three to 5.3 per 100,000 persons from 2000 to 2017 [1]. Adult foreign body ingestion is most commonly observed in patients with psychiatric disorders, developmental delays, alcohol intoxication, edentulous status, and incarcerated individuals seeking secondary gain [2, 3]. Cases of adult ingestions usually include a history of swallowing an object and an acute onset of symptoms like dysphagia or odynophagia. Radiographic imaging is critical in differentiating between foreign body ingestions and other conditions with similar presenting symptoms. Radiographs also assist in the localization of the object and, as in our case, sometimes the identification of the object. Cases of low-risk ingestions of small, blunt objects can be monitored clinically and do not require endoscopic evaluation, though 10% to 20% of ingestions are high risk and require intervention [3]. High-risk ingestions include ingestion of sharp objects, batteries, or magnets, or those causing complete obstruction. Interventions, endoscopic or surgical, decrease the risk of complications, including aspiration, obstruction, ulceration, perforation, and death. This case will highlight the presentation, evaluation, and management of a high-risk foreign body ingestion in an adult patient.

## Case presentation

A 55-year-old male patient with a past medical history of diabetes, hyperlipidemia, and coronary artery disease presented to an internal medicine clinic with acute odynophagia. Two days prior to the presentation, the patient reported he was at a wedding and drank heavily at dinner. A few hours after the meal, the patient began to complain of “something feeling stuck” in his throat and pain with swallowing. He assumed he had swallowed a fish bone at dinner. Over the next two days, the patient tried eating various foods, such as rice, to help with the foreign body sensation. However, nothing seemed to help, prompting his clinic visit. The patient denied fevers, chills, nausea, vomiting, hematemesis, melena, or abdominal pain. 

**Figure 1:**
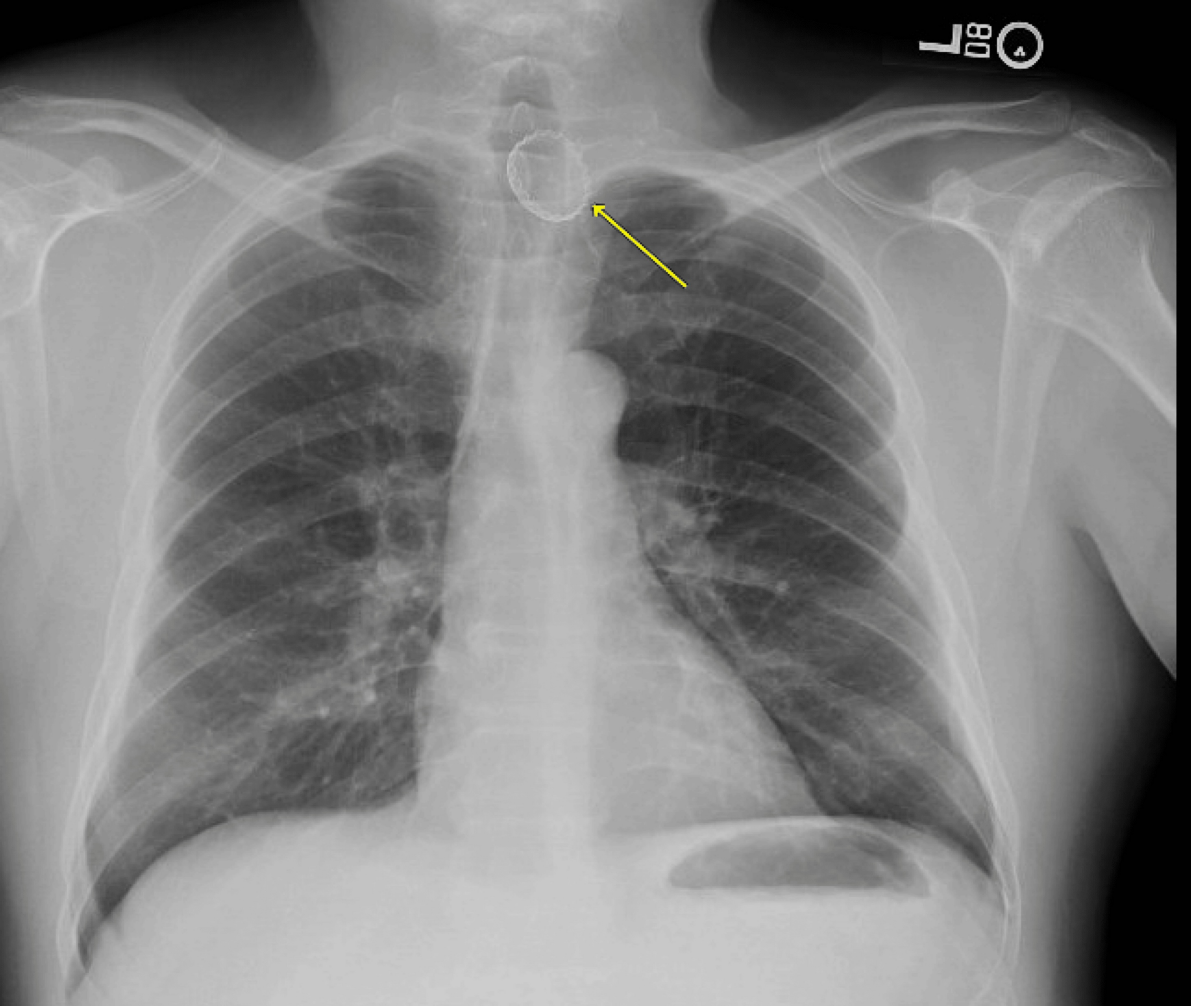
The patient's chest X-ray (posteroanterior (PA) view) A radiopaque structure projects over the upper thorax at the level of the thoracic inlet, presumably in the proximal esophagus.

**Figure 2:**
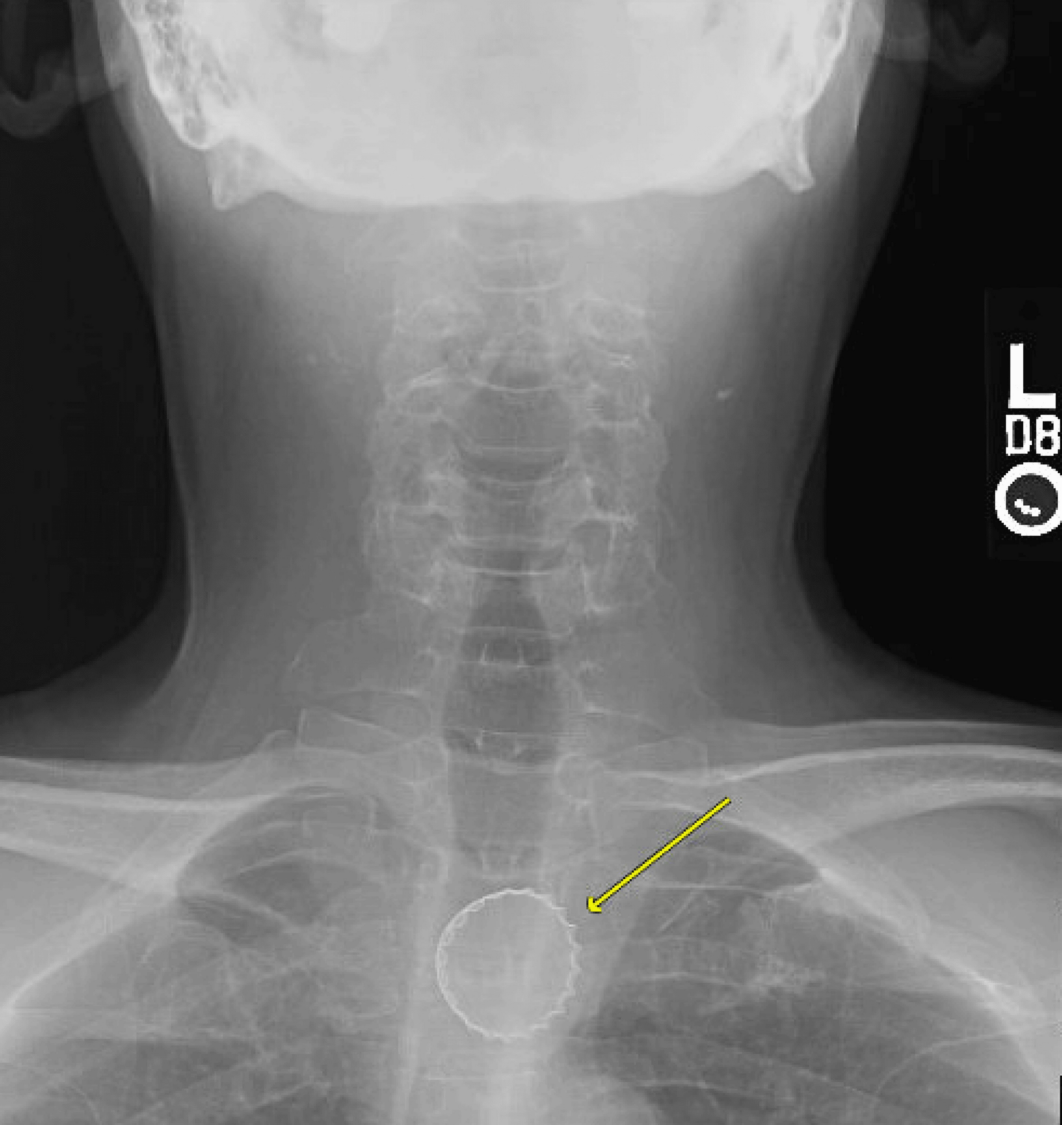
An X-ray of the cervical spine (anteroposterior (AP) view) A radiopaque circular body projecting over the upper esophagus was noted.

Vital signs were a temperature of 98.2 degrees F, a heart rate of 90 beats per minute, a respiratory rate of 18 breaths per minute, a blood pressure of 117/75 mm Hg, and an oxygen level of 98% on room air. The physical exam was notable for a comfortable middle-aged man, without drooling or respiratory distress. He had a normal oropharyngeal examination and a clear chest without stridor. X-rays of the chest and cervical spine were performed (Figures 1, 2). Findings were notable for a radiopaque circular structure with jagged edges projecting over the upper thorax, concerning for a metal bottle cap, presumably in the proximal esophagus. 

The patient was immediately sent to the emergency department. Gastroenterology was consulted, and the patient underwent an endoscopy. A foreign object was seen at 19 cm from the incisors within the wall of the proximal cervical esophagus with accompanying edema. Multiple attempts at removing the object were unsuccessful due to the bottle cap being lodged in the wall of the esophagus. Ear, Nose, and Throat (ENT) and Thoracic Surgery were consulted. Endoscopy was emergently performed with endotracheal intubation with gastroenterology and ENT. After multiple attempts, using a combination of Raptor forceps, direct visualization graspers, jaw thrust, neck extension with shoulder roll, and deflation of the endotracheal tube, a metallic bottle cap was retrieved. The patient was also found to have two superficial ulcerations. The patient had a nasogastric tube placed for esophageal rest and was placed on intravenous fluids and intravenous pantoprazole twice a day. The patient was successfully extubated. On hospital day 2, nasogastric feeds were initiated. On hospital day 3, an esophagram was performed, which was negative for perforation. The patient’s nasogastric tube was removed, and his diet was advanced to a soft diet. On hospital day 5, the patient was discharged with sucralfate for two weeks and pantoprazole twice daily for eight weeks. The patient was seen for follow-up by both Internal Medicine and Gastroenterology. The patient was asymptomatic during these visits and recovered without complications. 

## Discussion

This case highlights the importance of prompt evaluation and management to decrease the risk of complications from high-risk ingestions. Presentation of ingestions usually includes acute onset of dysphagia, odynophagia, retrosternal pain, vomiting, or the sensation of a foreign body. Certain patients, such as pediatric, elderly, or mentally impaired, may be unable to vocalize their symptoms. Presenting symptoms in these patients can include choking, refusal to eat, vomiting, abdominal pain, drooling, wheezing, blood-stained saliva, or respiratory distress [3]. In cases of tracheal compression by a foreign body or aspiration of saliva, patients may experience choking, dyspnea, or stridor [4]. According to both the American Society of Gastrointestinal Endoscopy (ASGE) and the European Society of Gastrointestinal Endoscopy (ESGE), the presence of drooling and the inability to swallow liquids is concerning for complete esophageal obstruction [4]. Physical examination is a critical part of the initial evaluation to detect complications. Fever, tachycardia, peritonitis, swelling of the neck or chest, or subcutaneous crepitus can all indicate perforation. A pulmonary exam may reveal stridor in partial obstructions, while wheezing, crackles, or rhonchi can point to aspiration [4]. 

In addition to a thorough history and physical, imaging plays a critical role in distinguishing foreign body ingestions from other conditions with similar symptoms, like achalasia, esophageal spasm, esophageal mass, Zenker’s diverticulum, esophageal strictures, reflux esophagitis, candida esophagitis, eosinophilic esophagitis, extrinsic mass, or certain musculoskeletal conditions. The ESGE recommends plain radiographs to confirm the presence, location, size, configuration, and number of foreign bodies, especially if the type of foreign body is unknown [4]. It is important to note that radiolucent objects are rarely seen on plain radiographs. This includes food bolus, fish or chicken bones, wood, plastic, glass, and thin metal objects [2, 5, 6]. The ESGE does not recommend barium swallow because of aspiration risk and increased difficulty with endoscopic evaluation after barium ingestion [4]. Computed tomography (CT) scan is recommended for patients suspected of perforation or small bowel obstruction [4,6]. In our patient, given the uncertainty of which foreign body was ingested, X-ray was crucial to determine management. Although his symptoms were relatively mild, the discovery of the bottle cap escalated his perforation risk, and emergent endoscopy was needed. This case highlights the importance of plain radiographs for risk triaging foreign body ingestion, particularly when the object in question is unknown. Radiographs can also be useful for serial imaging to track movement of radiopaque foreign bodies and detect complications, including pneumomediastinum or pneumoperitoneum due to pharyngeal or upper gastrointestinal perforations. Lateral radiographs can also detect widening of prevertebral soft tissue, suggesting edema [6].

Management varies depending on symptoms, the object ingested, radiographic findings, and complications at the time of presentation. Most cases of foreign body ingestion can be monitored clinically, though 10% to 20% require intervention to decrease complications, including impaction, ulceration, perforation, and death [3]. Most interventions include an endoscopic approach with esophagogastroduodenoscopy, with less than 1% requiring surgical intervention [7]. Indications for surgical intervention include perforation, bleeding that cannot be treated endoscopically, unsuccessful endoscopic retrieval, foreign body out of endoscopic reach, or small bowel obstruction [4, 7]. Complete esophageal obstruction and ingestion of batteries and sharp or pointed objects are reasons for emergent endoscopy, ideally within two hours [2, 4, 7]. Sharp or pointed foreign bodies, such as those found in our patient, can result in perforation leading to mediastinitis or peritonitis [7]. Batteries lodged in the esophagus may cause necrosis from pressure on the mucosa, release of alkaline substances causing liquefactive necrosis, fistula formation, mercury poisoning, and perforation; thus, prompt removal is necessary [2,4,7]. Urgent therapeutic endoscopy, within 24 hours, is recommended for foreign body ingestions without complete obstruction [4]. However, 80% of foreign body ingestions usually do not require endoscopic evaluation. The ASGE and ESGE both recommend that asymptomatic cases of ingestion of small, blunt objects, apart from batteries and magnets, may be monitored in the outpatient setting. Spontaneous passage usually occurs within four to six days, though in rare cases it can take up to four weeks [7]. In these cases, X-rays can be used weekly to track the foreign body. Once the foreign body passes the ileocecal valve, spontaneous passage is expected, and colonoscopic intervention is generally not indicated [7]. 

## Conclusions

Prompt clinical evaluation, triaging, appropriate use of imaging, and urgent specialist consultation in certain cases are all critical in the effective management of foreign body ingestion. Most cases of blunt, low-risk objects can be observed in an outpatient setting. However, urgent referral to the emergency department for prompt intervention is crucial in cases of suspected perforation, airway compromise, or sharp or corrosive objects like batteries or magnets. Plain radiographs are important in localizing and potentially identifying foreign bodies and guiding management decisions and interventions to minimize complications. Overall, a thorough understanding of presentation, diagnostic options, and intervention guidelines is essential in treating cases of foreign body ingestion.
